# The Silent Cost of Gender in Mitral Valve Surgery: A Propensity-Score Matched Analysis

**DOI:** 10.1093/ejcts/ezaf451

**Published:** 2025-12-14

**Authors:** Leo Pölzl, Ronja Lohmann, Clemens Engler, Maria Ioannou-Nikolaidou, Felix Nägele, Jakob Hirsch, Michael Graber, Vanessa Heim, Sophia Schmidt, Ludwig Müller, Daniel Höfer, Johannes Holfeld, Lena Tschiderer, Michael Grimm, Nikolaos Bonaros, Can Gollmann-Tepeköylü

**Affiliations:** Department of Cardiac Surgery, Medical University of Innsbruck, 6020 Innsbruck, Austria; Department of Cardiac Surgery, Medical University of Innsbruck, 6020 Innsbruck, Austria; Institute of Clinical and Functional Anatomy, Medical University of Innsbruck, 6020 Innsbruck, Austria; Department of Cardiac Surgery, Medical University of Innsbruck, 6020 Innsbruck, Austria; Department of Cardiac Surgery, Medical University of Innsbruck, 6020 Innsbruck, Austria; Institute of Clinical and Functional Anatomy, Medical University of Innsbruck, 6020 Innsbruck, Austria; Department of Cardiac Surgery, Medical University of Innsbruck, 6020 Innsbruck, Austria; Department of Cardiac Surgery, Medical University of Innsbruck, 6020 Innsbruck, Austria; Department of Cardiac Surgery, Medical University of Innsbruck, 6020 Innsbruck, Austria; Department of Cardiac Surgery, Medical University of Innsbruck, 6020 Innsbruck, Austria; Department of Cardiac Surgery, Medical University of Innsbruck, 6020 Innsbruck, Austria; Department of Cardiac Surgery, Medical University of Innsbruck, 6020 Innsbruck, Austria; Department of Cardiac Surgery, Medical University of Innsbruck, 6020 Innsbruck, Austria; Department of Cardiac Surgery, Medical University of Innsbruck, 6020 Innsbruck, Austria; Institute of Clinical Epidemiology, Public Health, Health Economics, Medical Statistics and Informatics, Medical University of Innsbruck, 6020 Innsbruck, Austria; Department of Cardiac Surgery, Medical University of Innsbruck, 6020 Innsbruck, Austria; Department of Cardiac Surgery, Medical University of Innsbruck, 6020 Innsbruck, Austria; Department of Cardiac Surgery, Medical University of Innsbruck, 6020 Innsbruck, Austria

**Keywords:** mitral valve disease, mitral valve surgery, minimal invasive surgery, sex-differences

## Abstract

**Objectives:**

Mitral valve (MV) disease shows sex-specific differences in morphology and outcomes. Women often present later, undergo replacement more frequently, and experience worse survival. This study investigated sex-related disparities in surgical approach, repair rates, and outcomes of MV surgery.

**Methods:**

A total of 1531 consecutive patients undergoing MV surgery with or without concomitant tricuspid valve procedure were analysed retrospectively. Baseline characteristics, operative strategies, and outcomes were compared between sexes. Propensity score matching was used to adjust for baseline differences. Primary outcomes were 30-day and 5-year mortality. Baseline and procedural characteristics, including morphology, repair rates, use of minimally invasive MV surgery (MIMVS), and concomitant tricuspid disease, were compared between groups.

**Results:**

Female patients (44%) were older (68 vs 62 years, *P* < .001), more symptomatic (New York Heart Association [NYHA] III: 60% vs 46%, *P* < .001), and more likely to have annular calcification (15% vs 5%, *P* < .001) or concomitant tricuspid disease (25% vs 36%, *P* < .001). Carpentier type IIIa was more prevalent in women (21% vs 4%), while type II predominated in men (75% vs 49%). MIMVS and repair were less frequent in women (49% vs 65% and 67% vs 85%, both *P* < .001). Female sex was associated with increased 30-day (HR 4.07, 95% CI 1.51-11.0; *P* = .006) and 5-year mortality (HR 1.58, 1.02-2.46; *P* = .043). After adjusting for morphology and calcification, sex was no longer an independent predictor of repair rates or long-term mortality.

**Conclusions:**

Women present at a later stage of the disease and with more complex MV pathology, resulting in lower repair and MIMVS rates and higher perioperative mortality. These disparities are largely attributable to disease morphology rather than sex *per se*. Earlier referral of women is essential to improve outcomes.

## Graphical abstract

**Figure:**
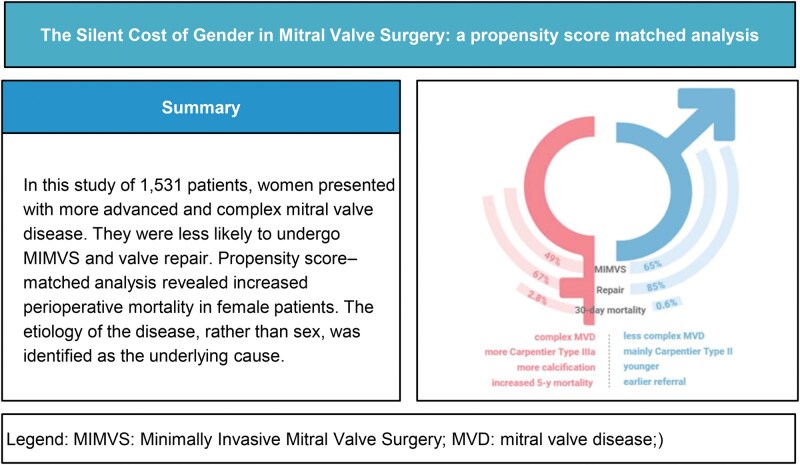

## Background

Significant sex-based disparities persist in the diagnosis, treatment, and outcomes of cardiovascular diseases, including mitral valve (MV) disorders.^1^^,^^2^ Differences can be observed in both valve morphology and disease pathology, with women more frequently presenting with bileaflet prolapse and leaflet thickening. Differences in calcification patterns are also evident, as annular calcification occurs more commonly in women.^3–5^ Each of these factors may influence treatment strategies and outcomes, contributing to higher rates of MV replacement and increased postoperative mortality.^5^ Additionally, women remain underdiagnosed, understudied, and undertreated in cardiology, leading to delayed interventions and worse prognoses.^6^^,^^7^

In many cases of MV disease, primary mitral regurgitation, and mitral stenosis, surgery remains the only treatment option.^8^ Minimally invasive MV surgery (MIMVS) has emerged as a transformative approach for treating MV disease, offering benefits such as reduced surgical trauma, shorter hospital stays, and faster recovery compared to conventional open-heart surgery.^9–12^ Sex-specific differences in the MIMVS have not been investigated yet.

This study aims to analyse sex-specific differences in (1) the morphology and extent of MV disease, (2) type (use of MIMVS, repair vs replacement), and (3) outcomes of intervention in patients referred for MV surgery in a tertiary university hospital. Furthermore, we aim to elucidate the underlying reasons for these treatment disparities.

By shedding light on these disparities, the study seeks to contribute to more equitable and personalized approaches in cardiac surgery, ultimately improving patient care for both males and females.

## Methods

### Study population and data collection

A consecutive series of patients undergoing cardiac surgery between 2010 and 2024 at the Medical University of Innsbruck were included in this study (*n* = 11 075). The indication for surgery was decided upon heart team discussion between cardiologists and cardiac surgeons. Only patients who underwent isolated MV surgery, with or without tricuspid valve intervention, were included in the final study cohort (*n* = 1531). The decision-making between repair or replacement was based on the preoperative anatomical and functional valve evaluation by transoesophageal echocardiography and cardiac CT. Mitral valve repair was the primary goal in all patients with suitable anatomy of a durable result. Relevant factors included valve pathology according to the Carpentier classification determined during surgical valve analysis, the presence of annular or leaflet calcifications, the degree of stenosis, and the probability of a durable repair. The surgical technique for repair (annuloplasty alone, additional use of artificial chordae, leaflet resection) was decided by the surgeon after inspection of the valve. The type of prosthesis used (mechanical or biological) was tailored to the existing recommendations of major scientific societies and the characteristics and wish of the informed patient. The use of minimally invasive techniques started at 2001 and underwent several refinements and evolution including the introduction of a quality improvement programme accompanying the transition to 3D-totally endoscopic procedures.^9^ Follow-up data were obtained by chart review and patient interviews and was 100% complete. Mortality data were obtained by the Austrian federal statistics institute (“*Statistik Austria*”) with a median follow-up of 5.0 [25th-75th percentile 2.25-5.0] years. This study was performed in accordance with the Declaration of Helsinki, and permission to use anonymized data without patient consent for this study was obtained from the Innsbruck Medical University Institutional Review Board (IRB number *1203/2019*).

### Primary outcome and statistical analysis

The primary end-point of this study was all-cause mortality within 30 days and 5 years after surgery. Secondary outcomes included the underlying MV morphology, which was classified de novo based on operative reports rather than transferred from existing records, as well as the presence of calcifications and the requirement for concomitant procedures. Procedure-related details (surgical access, repair, and replacement rates) and postoperative outcomes, such as length of intensive care unit (ICU) stay, occurrence of acute kidney injury requiring ultrafiltration, and the need for extracorporeal membrane oxygenation (ECMO), were also evaluated. Mortality during follow-up was additionally assessed to provide long-term outcome data. Categorical variables are presented as frequencies and proportions. Continuous variables are presented as medians and 25th-75th percentiles. Differences between groups were assessed by Chi-squared, Fisher’s exact, and Wilcoxon rank sum test as appropriate. Cox proportional hazards regression was used to estimate hazard ratios (HRs) and corresponding 95% confidence intervals (CIs). Logistic regression was performed to estimate the odds ratio (OR) and corresponding 95% CI. Sex was included as a covariate in the regression models to compare outcomes between women and men. Models were adjusted for the EuroSCORE II reflecting preoperative comorbidities. Five-year mortality analysis was performed after propensity score matching of women and men using nearest neighbour matching. Variables were chosen based on differences between women and men as well as their clinical significance (calliper 0.1; matching variables: age, New York Heart Association [NYHA], history of myocardial infarction [MI], history of stroke, history of smoking). Kaplan-Meier estimates were used to plot survival curves and compared using log-rank test. Utilization of MIMVs and repair rates were plotted in a bar chart according to Carpentier classification, separately by sex and combined for all patients.

To assess the robustness of our findings, we performed a sensitivity analysis after excluding patients who underwent concomitant TV procedures. *P*-values < .05 were considered statistically significant. All statistical analyses were performed with R Version 4.4.3.

## Results

### Sex-based differences in baseline characteristics prior to MV surgery

Between 2010 and 2024, a total of 1531 patients underwent isolated MV surgery at our centre, including those who had concomitant tricuspid valve surgery if clinically indicated; 44.2% of patients were female (**Table 1**). Females were significantly older than males (68 vs 62 years; *P *< .001). They were less likely to have a history of smoking (15% vs 23%; *P *< .001), MI (1.9% vs 4.6%; *P *= .005) and percutaneous coronary intervention (PCI) (2.5% vs 4.7%; *P *= .026), but more likely to have a history of stroke (7.2% vs 3.9%; *P *= .004). Left ventricular ejection fraction did not differ between sexes (60 vs 60%; *P* = .500). However, female patients were more symptomatic, with a significantly higher proportion presenting in NYHA class III (60% vs 46%; *P* < .001 for NYHA distribution). In line, significantly higher NT-proBNP levels were observed in women (847 vs 376 ng/L; *P *< .001). EuroSCORE II, which reflects overall preoperative comorbidities, was significantly higher in females compared to males (2.8% vs 1.3%; *P *< .001).

**Table 1.** Patient Characteristics

Characteristics	Female	Male	*P*-value
	*N *= 677	*N* = 854	
Age (years)	68 (58, 74)	62 (53, 71)	**<.001**
BMI	23.3 (21.0, 27.0)	25.0 (23.0, 27.7)	**<.001**
Diabetes	64 (9.5%)	68 (8.0%)	.300
Dyslipidaemia	345 (51%)	419 (49%)	.500
Hypertension	436 (64%)	516 (60%)	.110
History of smoking	103 (15%)	196 (23%)	**<.001**
Creatinine (mg/dL)	0.88 (0.76, 1.04)	1.03 (0.91, 1.18)	**<.001**
On dialysis	6 (1.0%)	8 (1.0%)	>.900
COPD	65 (9.6%)	64 (7.5%)	.140
History of stroke	49 (7.2%)	33 (3.9%)	**.004**
History of MI	13 (1.9%)	39 (4.6%)	**.005**
History of PCI	17 (2.5%)	40 (4.7%)	**.026**
LV-EF (%)	60 (54, 64)	60 (53, 65)	.500
LV-EF groups			.200
<20%	1 (0.1%)	1 (0.1%)	
21%-30%	3 (0.4%)	12 (1.4%)	
31%-50%	134 (20%)	176 (21%)	
>50%	535 (79%)	660 (78%)	
NYHA			**<.001**
I	43 (7.5%)	127 (17%)	
II	147 (26%)	251 (33%)	
III	346 (60%)	344 (46%)	
IV	38 (6.6%)	32 (4.2%)	
NT-proBNP (ng/L)	847 (336, 1898)	376 (125, 1358)	**<.001**
EuroSCOREII	2.80 (1.50, 5.30)	1.30 (0.75, 2.70)	**<.001**

*n* (%); median (Q1, Q3).

Abbreviations: BMI: body mass index; COPD: chronic obstructive pulmonary disease; LV-EF: left ventricular ejection fraction; MI: myocardial infarction; NYHA: New York Heart Association; PCI: percutaneous coronary intervention.

The bold values indicate statistical significance (*p* < 0.05).

### Morphology of MV disease differs between women and men

The underlying MV pathology, classified according to the Carpentier classification, differed between women and men. While type II was present in 75% of men and only 49% of women, type IIIa was observed in 3.9% of men but in 21% of women (*P* < .001). Mitral valve prolapse was more commonly located in the posterior leaflet (PML) and did not differ significantly between sexes (male vs female: anterior mitral leaflet [AML]: 12% vs 18%; PML: 76% vs 71%; *P* = .11). Barlow’s disease was diagnosed in both sexes, with no significant difference (men: 10% vs women: 8.1%; *P* = .2). However, the prevalence of MV calcification was higher in women compared with men (15% vs 5%, *P* < .001) (all **Table 2**).

**Table 2.** Aetiology of Mitral Valve Disease

Characteristics	Female	Male	*P*-value
	*N* = 677	*N *= 854	
Carpentier classification			**<.001**
I	128 (19%)	95 (11%)	
II	323 (49%)	635 (75%)	
IIIa	142 (21%)	33 (3.9%)	
IIIb	15 (2.3%)	13 (1.5%)	
Mixed	17 (2.6%)	18 (2.1%)	
Unknown	36 (5.4%)	51 (6.0%)	
Location of MV prolapse			.110
AML	57 (18%)	77 (12%)	
PML	224 (71%)	479 (76%)	
Commissures	2 (0.6%)	5 (0.8%)	
Multisegmental	33 (10%)	71 (11%)	
Mb. Barlow	55 (8.1%)	87 (10%)	.200
MV calcification			**<.001**
None	577 (85%)	811 (95%)	
Leaflet	20 (3.0%)	8 (0.9%)	
Annulus	39 (5.8%)	21 (2.5%)	
Combination or not specified	41 (6.0%)	14 (1.7%)	

*n* (%).

Abbreviations: AML: anterior mitral leaflet; MV: mitral valve; PML: posterior mitral leaflet.

The bold values indicate statistical significance (*p* < 0.05).

### Minimally invasive approaches are more commonly used in male patients

**Figure 1.**
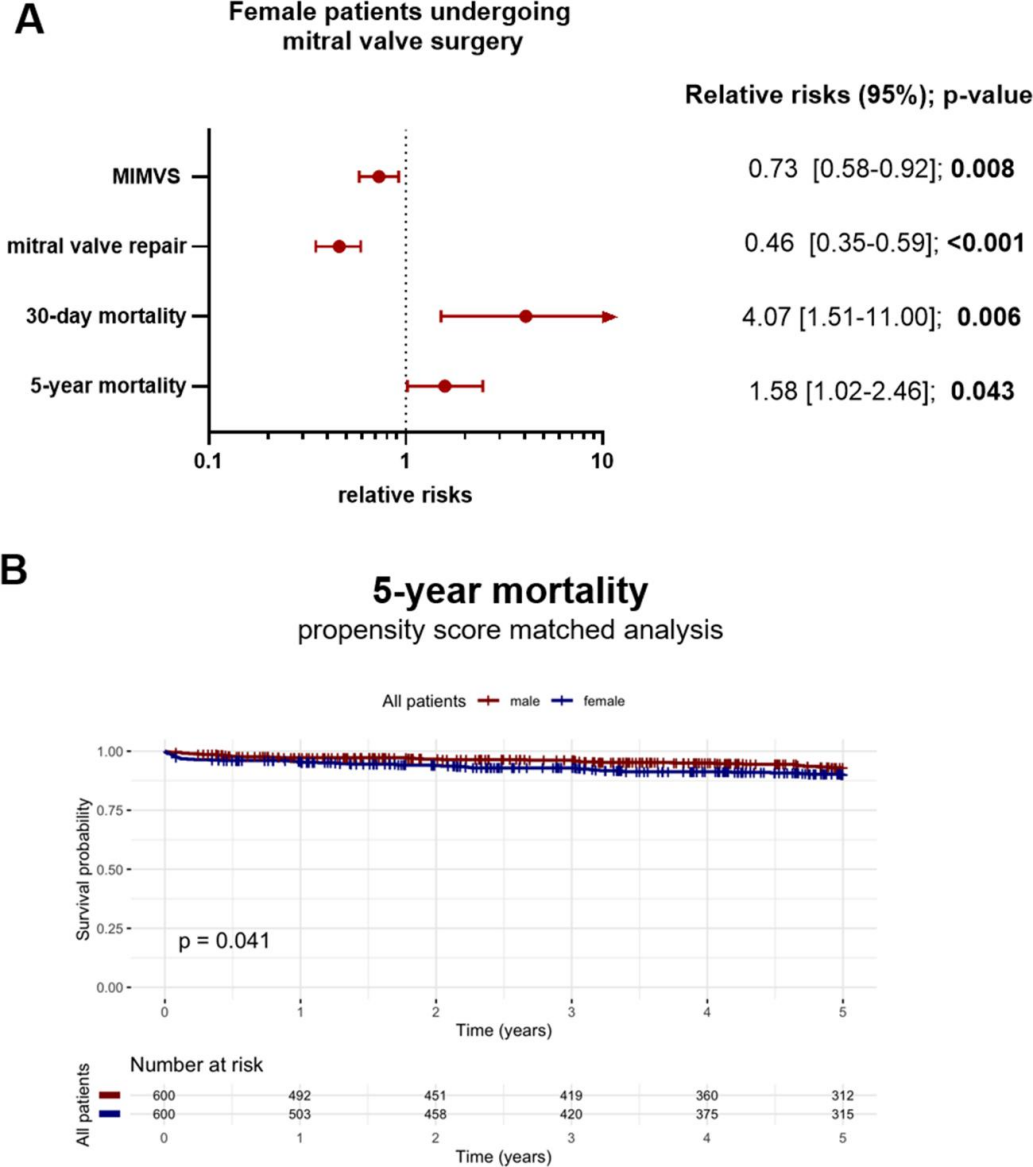
Female Patients Exhibit a Less Favourable Postoperative Outcome. (A) Risk of female patients was compared with male patients. Models were adjusted for EuroSCORE II reflecting all preoperative comorbidities. For MIMVS and mitral valve repair, a logistic regression model was performed, and the measure of association is presented as odds ratio. For 30-day mortality and 5-year mortality, a Cox proportional hazards regression was performed, and the measure of association is presented as hazard ratio. (B) Females and males were propensity-score matched based on preoperative characteristics using a calliper of 0.1. Kaplan-Meier curves were obtained and the log-rank test was performed to assess 5-year mortality. Abbreviation: MIMVS: minimally invasive mitral valve surgery.

The urgency of surgery did not differ significantly between men and women (elective procedure: 94% vs 93%). However, the surgical access strategy differed markedly between sexes (*P* < .001). A minimally invasive (MIMVS) approach was used in 65% of men, compared to only 49% of women. Among the remaining patients, full sternotomy was performed in 22% of men and 29% of women, while partial sternotomy was used in 13% of men and 22% of women. Mitral valve repair was achieved in 85% of male patients but only in 67% of female patients; therefore, MV replacement was significantly more frequent in women (*P* < .001). Even after adjusting for all preoperative comorbidities, regression analysis revealed that females were unlikely to receive MIMVS (OR 0.73; 95% CI 0.58-0.92; *P*-value = .008) and repair (OR 0.46; 95% CI 0.35-0.59; *P*-value < .001) (**Figure 1A**). Concomitant tricuspid valve surgery was performed significantly more often in women (36%) compared to men (25%). Cross-clamp time (CCT) and total perfusion time was longer in male patients (102 vs 98 min; *P*-value = .038; perfusion time: 172 vs 163 min, *P*-value <.001) (**Table 3**).

**Table 3.** Surgical Characteristics and Perioperative Outcome

Characteristics	Female	Male	*P*-value
	*N* = 677	*N* = 854	
Elective operation	618 (93%)	789 (94%)	.900
Approach			**<.001**
Full sternotomy	199 (29%)	187 (22%)	
Partial sternotomy	148 (22%)	108 (13%)	
MIMVS	330 (49%)	559 (65%)	
MV procedure			**<.001**
Replacement	222 (33%)	131 (15%)	
Repair	455 (67%)	723 (85%)	
Concomitant TV procedure	243 (36%)	214 (25%)	**<.001**
Total CCT (minutes)	98 (80, 121)	102 (82, 124)	**.038**
Total perfusion time (minutes)	163 (134, 200)	172 (144, 211)	**<.001**
Perioperative outcome			
ICU stay duration (days)	0.0 (0.0, 2.0)	0.0 (0.0, 1.0)	**.020**
Prolonged ICU stay (>1 day)	200 (30%)	200 (23%)	**.007**
ECMO	17 (2.5%)	23 (2.7%)	.800
Ultrafiltration	65 (9.6%)	70 (8.2%)	.300
30-day mortality	19 (2.8%)	5 (0.6%)	**<.001**

*n* (%); median (Q1, Q3).

Abbreviations: CCT: cross-clamp time; ECMO: extracorporeal membrane oxygenation; ICU: intensive care unit; MIMVS: minimally invasive mitral valve surgery; MV: mitral valve.

The bold values indicate statistical significance (*p* < 0.05).

### Female patients exhibit a less favourable perioperative outcome

Female patients required significantly longer postoperative stays in the ICU (*P* = .007). No significant differences were observed between sexes regarding the need for ECMO or ultrafiltration. However, 30-day mortality was notably higher in women at 2.8%, compared to 0.6% in men (*P* < .001) (see **Table 3**). Even after adjustment for all preoperative comorbidities, women had an increased risk of 30-day mortality following surgery (HR 4.07 95% CI 1.51-11.0; *P* = .006) (**Figure 1A**). After propensity score matching, 5-year mortality was significantly higher in female patients (log-rank *P* = .041). Consistently, the multivariable Cox regression model also demonstrated an increased risk in women (HR: 1.58; 95% CI: 1.02-2.46; *P* = .043) (**Figure 1A and B**).

### Sex-specific differences in MV morphology cause decreased repair rates and poorer long-term outcome

**Figure 2.**
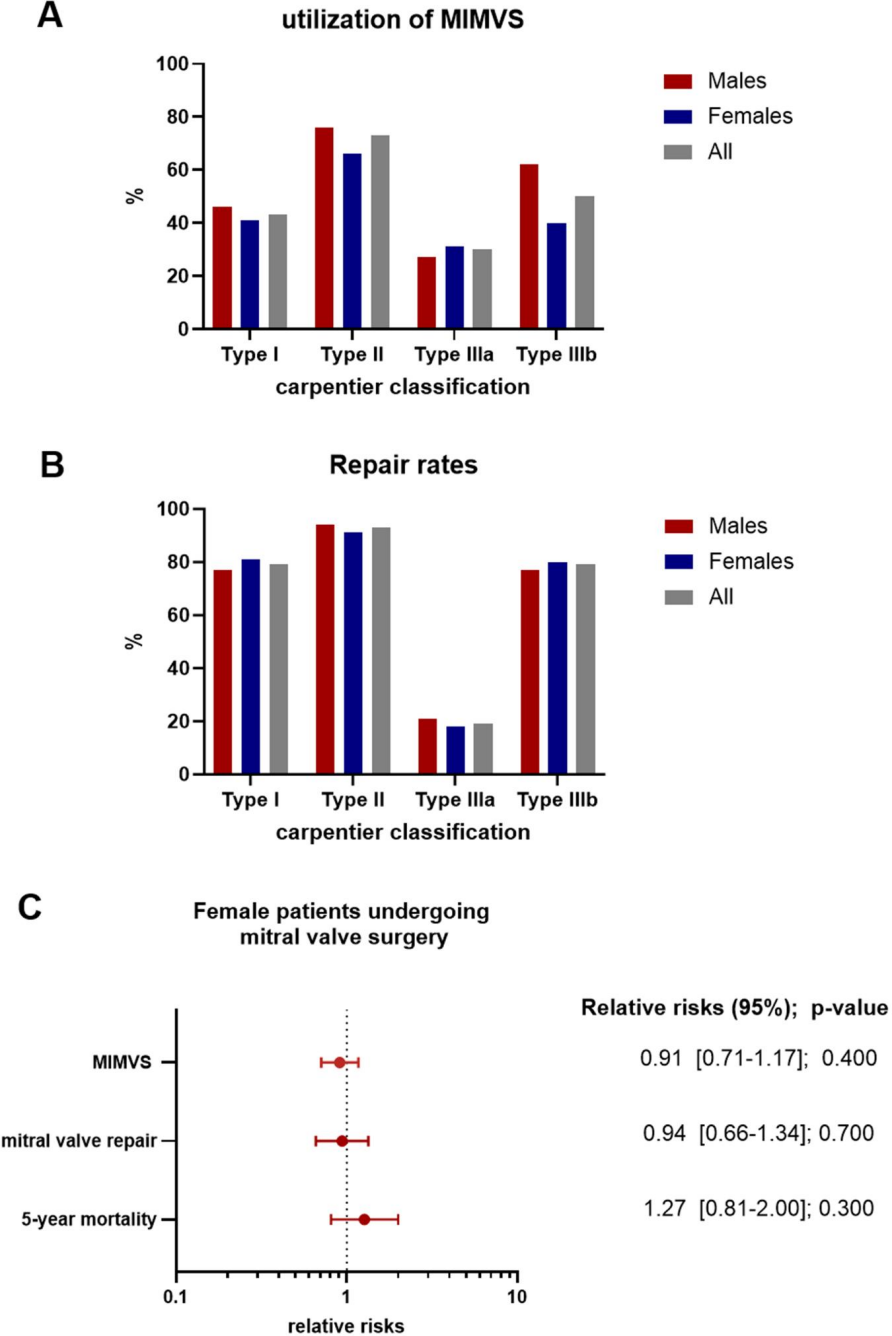
Sex-specific Differences in Aetiology Cause Decreased Repair Rates and Poorer Long-term Outcome. (A) Utilization of MIMVS varied significantly across Carpentier classification categories but was similar between sexes. (B) Repair rates varied significantly across Carpentier classification categories but was similar between sexes. (C) Risk of female patients was compared with male patients. The logistic regression model for MIMVS was adjusted for Carpentier classification and EuroSCORE II. The logistic regression model for mitral valve repair was adjusted for Carpentier classification, presence of calcification, and EuroSCORE II. Both are presented as odds ratios. For 5-year mortality, a Cox proportional hazards regression adjusted for valve repair was performed and is presented as hazard ratio. After adjustment, female sex was no longer identified as an independent risk factor. Abbreviation: MIMVS: minimally invasive mitral valve surgery.

MIMVS and replacement rates differed significantly across Carpentier classification groups. The use of MIMVS differed markedly by valve pathology, being more common in Carpentier type II disease (73%) than in type IIIa disease (30%) (**Figure 2A** and **Table S1**). Similar, while MV repair was performed in 93% of patients with Carpentier type II disease, only 19% of patients with type IIIa pathology underwent repair. These differences were not sex-dependent (**Figure 2B** and **Table S1)**. In logistic regression models adjusted for Carpentier classification and EuroSCORE II, sex was not significantly associated with MIMVS (OR 0.91; 95% CI 0.71-1.17; *P* = .400). Furthermore, adjusted for Carpentier classification, valve calcification, and EuroSCORE II, female sex was not associated with repair rates (OR 0.94; 95% CI 0.66-1.34; *P* = .700). Similarly, no sex-specific differences in long-term outcomes were observed after adjusting for valve replacement. Female sex was no longer an independent risk factor for 5-year mortality (HR 1.27; 95% CI 0.81-2.00; *P* = .300) (**Figure 2C**).

## Discussion

Data on sex-based disparities in the treatment of MV disease remain limited. In this retrospective analysis of 1531 consecutive patients undergoing MV surgery, we observed marked differences between male and female patients:

- Female patients were older and more symptomatic at surgery.
- Women had a higher prevalence of calcification, tricuspid involvement and functionally more complex MV pathology.
- They underwent fewer repairs and minimally invasive surgeries.
- Female sex was associated with increased 30-day mortality and 5-year mortality.
- After adjusting for morphology and calcification, sex itself was not an independent predictor of long-term outcomes.

Women presented to surgery at an older age and in a more compromised clinical condition. Preoperative NT-proBNP levels were significantly higher in women, suggesting increased cardiac filling pressures. Consistently, women reported more severe symptoms compared to men. As surgeons, we can only speculate on the underlying causes of delayed referral. However, it is well-documented that cardiovascular disease is underdiagnosed and often diagnosed later in women than in men.^6^^,^^7^ Although not specific to valvular disease, this finding underscores the need for greater awareness of cardiovascular risk in female patients.

Intraoperatively, we observed significantly more MV calcification in female patients. This observation is in line with the existing literature.^3–5^ This could reflect either (1) a longer duration of disease before surgical intervention or (2) a distinct pathophysiology of MV degeneration in women. While most male patients presented with Carpentier type II (less complex) MV disease, women more frequently exhibited a more complex pathology of mitral regurgitation (Carpentier type IIIa). This highlights a fundamental difference in disease patterns between sexes. Although data on sex-specific pathophysiology in MV disease are scarce, one might hypothesize that the higher incidence of functional mitral disease in women could be partially explained by hormonal influences, such as oestrogen-related changes in extracellular matrix regulation.^3^^,^^5^^,^^13^^,^^14^ Interestingly, no differences in the incidence of Barlow’s disease was noted.

In female patients, tricuspid valve repair was more frequently required. This may be attributed to 2 main factors: (1) the aforementioned differences in morphology and pathogenesis of MV disease, or (2) the fact that women tend to be referred for surgery at a later stage, by which time secondary tricuspid valve disease may already be present. The latter is supported by the fact that females were presented with more significant symptoms and higher levels of NT-pro-BNP. Interestingly, in a multicentre study, the indication to perform tricuspid annuloplasty was more commonly followed in female than male patients.^15^ This fact underscores the fact that female patients are not subjected to undertreatment if concomitant tricuspid surgery is needed.

Minimally invasive MV surgery was performed in 65% of male patients, compared to only 49% of female patients. Several factors may account for this disparity. First, women present with more advanced disease and comorbidities, making full sternotomy a safer or more practical approach. Second, smaller body size and anatomical considerations in women may pose technical challenges to MIMVS. Regardless of the reasons, if MV repair via MIMVS is considered the gold standard in our institution, these findings suggest that women receive less optimal surgical treatment.^9–12^ Specific selection criteria such as peripheral calcifications precluding the use of remote access perfusion or mitral annular or leaflet calcifications which are considered to be important for safe MIMVS were meticulously followed. Both the older age and the higher incidence of calcifications of the mitral apparatus may have frequently influenced surgical decision-making towards a non-MIMVS approach in women. Importantly, women experienced significantly higher 5-year mortality following surgery. Even after adjusting for confounders using propensity score matching and multivariable regression models, female sex remained an independent risk factor for long-term mortality.

To assess the risk of bias based on the pre-existing MV pathology, we performed a subgroup analysis within each Carpentier classification category. Hereby we observed that utilization of MIMVS and repair rates did not differ between sexes. Moreover, after adjustment for the Carpentier classification of MV disease and the presence of calcification, no sex-based differences in surgical access and repair rates were found. Similarly, after adjusting for valve replacement, no significant differences in long-term mortality between women and men were observed. Accordingly, the morphology of MV and the resulting need for valve replacement appears to be responsible for the poorer long-term outcomes, rather than female sex itself.

The minimally invasive programme expanded progressively over the study period with ongoing quality improvements, and although morbid adiposity was an early relative contraindication, body mass index (BMI) and concomitant tricuspid disease were not applied as exclusion criteria, making selection bias unlikely. Among patients who underwent MV replacement, prosthesis selection followed standard age-based practice, but the small number of replacements and low event rate precluded a meaningful comparison of outcomes between biological and mechanical valves.

Importantly, sensitivity analyses restricted to isolated MV procedures (**Tables S2  and  S3**, **Figure S1**) as well as to elective cases yielded results consistent with the main cohort, reinforcing that the inclusion of patients with concomitant tricuspid disease or non-elective presentations did not materially alter our conclusions.

These findings highlight that the higher long-term mortality observed in women is largely attributable to differences in underlying valve pathology and the resulting surgical procedures, rather than sex alone. They underscore a complex interplay of biological, anatomical, and possibly sociocultural factors that influence disease manifestation and treatment decisions. Addressing these disparities will require not only careful patient selection and surgical planning, but also further research into why women present with more advanced disease and complex pathology.

### Strengths and limitations

A key strength of this study is that it represents a consecutive series within a long-standing and experienced mitral programme, thereby minimizing potential bias from learning-curve effects. The large proportion of elective procedures (94%) further enhances the consistency of the cohort (**Table 3**). However, several limitations must be acknowledged. The retrospective design inherently carries the risk of unmeasured confounding. Female patients in the cohort were generally smaller in size, which may have influenced the surgical approach and the likelihood of undergoing MIMVS. In addition, the number of acute cases, such as infective endocarditis, was too small to allow statistically reliable conclusions for these subgroups. Only a small subset of patients (*n* = 17, 1.0%) were classified as having ischaemic mitral regurgitation, reflecting the exclusion of patients undergoing concomitant CABG. Future studies specifically addressing functional and ischaemic aetiologies are needed to clarify potential sex-related differences in this subgroup. Finally, although some patients had potential for longer follow-up beyond 5 years, the analysis was limited to this time frame due to the progressively smaller at-risk population and reduced statistical power. Moreover, the potential risk of mortality unrelated to the MV disease increases after 5 years, and it is different between the 2 sexes. Nevertheless, future studies should explore longer-term, sex-specific outcomes.

## Conclusion

Women undergoing MV surgery present later, with more complex disease, and consequently experience lower repair and MIMVS rates as well as higher perioperative mortality. These disparities are largely attributable to underlying valve morphology and delayed referral rather than sex *per se*, underscoring the need for earlier recognition and referral of female patients.

## Supplementary Material

ezaf451_Supplementary_Data

## Data Availability

The data supporting this study are available from the corresponding author upon reasonable request.
