# Supplementary material for: The Silent Cost of Gender in Mitral Valve Surgery: A Propensity-Score Matched Analysis
Source: Eur J Cardiothorac Surg. 2025 Dec 14;68(1):ezaf451. doi: 10.1093/ejcts/ezaf451 (PMC12771641; doi:10.1093/ejcts/ezaf451)
Supplement: ezaf451_Supplementary_Data [file ezaf451_supplementary_data.docx]

**Supplemental Materials:**

Supplemental Table 3: Rates of minimally invasive mitral valve surgery (MIMVS) and mitral valve repair in male and female patients, presented by Carpentier classification. Differences between Carpentier classes were evaluated using the Chi-square test.

| **MIMVS Rate** | **I** N = 223*^1^* | **II** N = 958*^1^* | **IIIa** N = 174*^1^* | **IIIb** N = 29*^1^* | **p-value***^2^* |
| --- | --- | --- | --- | --- | --- |
| All patients | 97 (43%) | 696 (73%) | 53 (30%) | 14 (48%) | **<0.001** |
| Male patients | 44 (46%) | 483 (76%) | 9 (27%) | 8 (62%) | **<0.001** |
| Female Patients | 53 (41%) | 213 (66%) | 44 (31%) | 6 (40%) | **<0.001** |
| **Repair Rate** |  |  |  |  |  |
| All patients | 177 (79%) | 893 (93%) | 32 (18%) | 23 (79%) | **<0.001** |
| Male patients | 73 (77%) | 599 (94%) | 7 (21%) | 10 (77%) | **<0.001** |
| Female Patients | 104 (81%) | 294 (91%) | 25 (18%) | 13 (81%) | **<0.001** |
| *^1^* n (%)  *^2^* Pearson’s Chi-squared test | | | | | |
|  | | | | | |

Supplemental Table 2: Patient characteristics excluding patients undergoing concomitant TV procedure

| **Characteristics** | **Male** N = 640*^1^* | | **Female** N = 434*^1^* | | **p-value***^2^* |  |
| --- | --- | --- | --- | --- | --- | --- |
| Age (years) | 60 (52, 68) | | 65 (55, 73) | | **<0.001** |  |
| BMI | 25.0 (23.0, 27.1) | | 23.0 (21.0, 27.0) | | **<0.001** |  |
| Diabetes | 42 (6.6%) | | 30 (6.9%) | | 0.8 |  |
| Dyslipidemia | 318 (50%) | | 209 (48%) | | 0.6 |  |
| Hypertension | 366 (57%) | | 262 (60%) | | 0.3 |  |
| History of smoking | 143 (22%) | | 74 (17%) | | **0.034** |  |
| Creatinine (mg/dl) | 1.02 (0.91, 1.16) | | 0.84 (0.73, 0.99) | | **<0.001** |  |
| On dialysis | 5 (0.9%) | | 1 (0.3%) | | 0.4 |  |
| COPD | 40 (6.3%) | | 41 (9.4%) | | 0.052 |  |
| History of stroke | 24 (3.8%) | | 28 (6.5%) | | **0.043** |  |
| History of MI | 30 (4.7%) | | 7 (1.6%) | | **0.007** |  |
| History of PCI | 28 (4.4%) | | 13 (3.0%) | | 0.2 |  |
| LV-EF (%) | 60 (55, 65) | | 60 (55, 65) | | 0.2 |  |
| LV-EF groups |  | |  | | 0.6 |  |
| <20% | 0 (0%) | | 1 (0.2%) | |  |  |
| 21-30% | 6 (0.9%) | | 2 (0.5%) | |  |  |
| 31-50% | 112 (18%) | | 76 (18%) | |  |  |
| >50% | 519 (81%) | | 352 (82%) | |  |  |
| NYHA |  | |  | | **<0.001** |  |
| I | 116 (20%) | | 37 (10%) | |  |  |
| II | 207 (36%) | | 110 (30%) | |  |  |
| III | 238 (41%) | | 192 (53%) | |  |  |
| IV | 20 (3.4%) | | 26 (7.1%) | |  |  |
| NT-proBNP (ng/l) | 223 (94, 793) | | 555 (238, 1,337) | | **<0.001** |  |
| EuroSCOREII | 1.00 (0.70, 1.80) | | 2.00 (1.10, 3.60) | | **<0.001** |  |
| *^1^* Median (Q1, Q3); n (%) | | | | | |  |
| *^2^* Wilcoxon rank sum test; Pearson’s Chi-squared test; Fisher’s exact test | | | | | |  |
| Supplemental Table 3: Surgical characteristics and perioperative outcome excluding patients undergoing concomitant TV procedure | | | | | |  |
| **Characteristics** | | **Male** N = 640*^1^* | | **Female** N = 434*^1^* | | **p-value***^2^* |
| Carpentier Classification | |  | |  | | **<0.001** |
| I | | 48 (7.6%) | | 56 (13%) | |  |
| II | | 507 (80%) | | 242 (57%) | |  |
| IIIa | | 18 (2.8%) | | 82 (19%) | |  |
| IIIb | | 9 (1.4%) | | 11 (2.6%) | |  |
| Mixed | | 14 (2.2%) | | 11 (2.6%) | |  |
| Unknown/not possible to define | | 38 (6.0%) | | 21 (5.0%) | |  |
| Location of MV prolapse | |  | |  | | 0.089 |
| AML | | 49 (9.7%) | | 38 (16%) | |  |
| PML | | 393 (78%) | | 173 (73%) | |  |
| Commissures | | 4 (0.8%) | | 2 (0.8%) | |  |
| Multisegmental | | 57 (11%) | | 23 (9.7%) | |  |
| MV Procedure | |  | |  | | **<0.001** |
| Replacement | | 80 (13%) | | 134 (31%) | |  |
| Repair | | 560 (88%) | | 300 (69%) | |  |
| Approach | |  | |  | | **<0.001** |
| Full sternotomy | | 97 (15%) | | 105 (24%) | |  |
| Partial sternotomy | | 69 (11%) | | 81 (19%) | |  |
| MIMVS | | 474 (74%) | | 248 (57%) | |  |
| MV Calcification | |  | |  | | **<0.001** |
| None | | 613 (96%) | | 376 (87%) | |  |
| Leaflet | | 6 (0.9%) | | 10 (2.3%) | |  |
| Annulus | | 14 (2.2%) | | 28 (6.5%) | |  |
| Combination or not specified | | 7 (1.1%) | | 20 (4.6%) | |  |
| Elective procedure | | 595 (94%) | | 389 (92%) | | 0.5 |
| Total CCT (min) | | 98 (79, 121) | | 94 (76, 116) | | **0.049** |
| Total perfusion time (min) | | 169 (141, 204) | | 158 (130, 195) | | **<0.001** |
| **Perioperative Outcome** | |  | |  | |  |
| ECMO | | 13 (2.0%) | | 10 (2.3%) | | 0.8 |
| Ultrafiltration | | 33 (5.2%) | | 31 (7.1%) | | 0.2 |
| ICU stay duration (days) | | 0.00 (0.00, 1.00) | | 0.00 (0.00, 1.00) | | **0.039** |
| ICU stay > 1d | | 111 (17%) | | 98 (23%) | | **0.033** |
| 30-day mortality | | 3 (0.5%) | | 9 (2.1%) | | **0.018** |
| *^1^* n (%); Median (Q1, Q3) | | | | | | |
| *^2^* Pearson’s Chi-squared test; Fisher’s exact test; Wilcoxon rank sum test | | | | | | |

Supplemental Figure 1: Survival analysis after excluding patients who underwent concomitant TV procedures, comparing male and female patients. Differences between groups were assessed using the log-rank test.

*
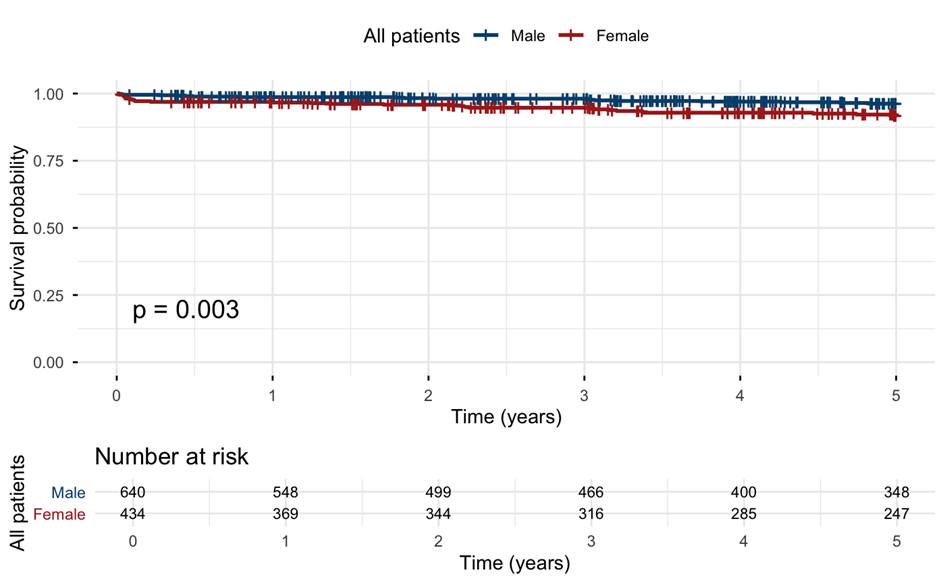
*
